# Phase Ib Study of Immunocytokine Simlukafusp Alfa (FAP-IL2v) Combined with Pembrolizumab for Treatment of Advanced and/or Metastatic Melanoma

**DOI:** 10.1158/2767-9764.CRC-24-0601

**Published:** 2025-02-24

**Authors:** Eva Munoz-Couselo, Ainara Soria Rivas, Shahneen Sandhu, Georgina V. Long, Miguel F. Sanmamed, Anna Spreafico, Elizabeth Buchbinder, Mario Sznol, Hans Prenen, Alexander Fedenko, Mohammed Milhem, Ana Maria Arance Fernandez, Jean-Jacques Grob, Lev Demidov, Caroline Robert, Christin Habigt, Stefan Evers, Nassim Sleiman, David Dejardin, Caroline Ardeshir, Nicole Martin, Christophe Boetsch, Jehad Charo, Volker Teichgräber, Anton Kraxner, Nino Keshelava, Oliver Bechter

**Affiliations:** 1 Vall d’Hebron University Hospital, VHIO, Vall d’Hebron Institute of Oncology, Barcelona, Spain.; 2 Department of Medical Oncology, University Hospital Ramón y Cajal, Madrid, Spain.; 3 Department of Medical Oncology, Peter MacCallum Cancer Centre and the University of Melbourne, Melbourne, Australia.; 4 Melanoma Institute Australia, The University of Sydney, Sydney, Australia.; 5 Faculty of Medicine and Health, The University of Sydney, Sydney, Australia.; 6 Department of Medical Oncology, Royal North Shore and Mater Hospitals, Sydney, Australia.; 7 Department of Medical Oncology, Clinica Universidad de Navarra, Pamplona, Spain.; 8 CIBERONC, Instituto de Salud Carlos III, Madrid, Spain.; 9 Division of Medical Oncology, Princess Margaret Cancer Centre University Health Network, Toronto, Canada.; 10 Beth Israel Deaconess Medical Center, Boston, Massachusetts.; 11 Yale University School of Medicine, New Haven, Connecticut.; 12 Antwerp University Hospital, Edegem, Belgium.; 13 P.A. Gertsen Moscow Oncology Research Institute, Moscow, Russia.; 14 University of Iowa, Iowa City, Iowa.; 15 Department of Oncology, Hospital Clínic of Barcelona and IDIBAPS, Barcelona, Spain.; 16 APHM and Aix-Marseille University, Marseille, France.; 17 N.N. Blokhin Russian Cancer Research Center, Moscow, Russia.; 18 Institut Gustave Roussy and Paris Saclay University, Villejuif, France.; 19 Roche Pharma Research and Early Development, Early Clinical Development Oncology, Roche Innovation Center Munich, Munich, Germany.; 20 Roche Pharma Research and Early Development, Early Clinical Development Oncology, Roche Innovation Center Basel, Basel, Switzerland.; 21 F. Hoffmann-La Roche Ltd, Welwyn Garden City, United Kingdom.; 22 Roche Pharma Research and Early Development, Early Clinical Development Oncology, Roche Innovation Center Zurich, Schlieren, Switzerland.; 23 Department of General Medical Oncology, University Hospitals Leuven, Leuven, Belgium.; 24 Department of Oncology, KU Leuven, Leuven, Belgium.

## Abstract

**Purpose:**

This study explored the combination of fibroblast activation protein (FAP) IL2 variant (FAP-IL2v), a novel immune-cytokine, with pembrolizumab in patients with advanced and/or metastatic melanoma.

**Patients and Methods:**

This open-label, multicenter, phase Ib clinical study (NCT03875079) evaluated the safety, tolerability, pharmacodynamics, pharmacokinetics, and antitumor activity of FAP-IL2v (simlukafusp alfa, RO6874281) in combination with pembrolizumab. Patients with advanced and/or metastatic melanoma were either checkpoint inhibitor (CPI)-naïve or CPI-experienced. Patients received 10 mg FAP-IL2v either continuously once every 3 weeks (Q3W) or in an induction/maintenance setting consisting of a 3-week induction phase with weekly (QW) dosing followed by continuous Q3W dosing. Pembrolizumab was dosed Q3W at 200 mg.

**Results:**

Eighty-three patients were treated: 16 patients in two safety run-in cohorts and 67 patients in two extension cohorts; 75 (90.4%) patients were CPI-experienced. The pharmacokinetics of FAP-IL2v in combination with pembrolizumab was similar to that after administration as monotherapy. Consistent with the proposed mode of action, FAP-IL2v preferentially expanded NK and CD8 T cells. The most common FAP-IL2v–related grade 3/4 adverse events were lymphopenia (23%), elevated γ-glutamyltransferase (8%), elevated alanine aminotransferase (6%), and infusion-related reaction (6%). A response was observed in 5 of 75 (6.7%) CPI-experienced patients (all partial responses) and 2 of 8 CPI-naïve patients (one complete response and one partial response). The median progression-free survival was 3.1 months.

**Conclusions:**

The safety profile of FAP-IL2v in combination with pembrolizumab was manageable and consistent with the known safety profile. However, further exploration of FAP-IL2v and pembrolizumab was precluded in patients with melanoma with prior CPI due to the lack of clinical activity.

**Significance:**

In this phase Ib study, the combination of FAP-IL2v, an immune-cytokine developed to overcome the limitations of wild-type IL2, with the CPI pembrolizumab did not show meaningful antitumor activity in patients who had progressed on prior CPI therapy, suggesting that FAP-IL2v alone cannot overcome CPI resistance or unresponsiveness.

## Introduction

Immunotherapy has transformed treatment outcomes for patients with metastatic melanoma, with 60% of patients achieving a sustained response (1). However, the unmet medical need remains high for metastatic melanoma, and additional treatment options are needed, particularly for patients who fail treatment with immune checkpoint inhibitors (CPI; ref. 2). Melanoma is an immunogenic tumor in which immunomodulatory treatments with therapeutic cytokines such as IL2 and IFN alfa 2 have shown benefit (3).

Fibroblast activation protein (FAP) IL2 variant (FAP-IL2v, also referred to as simlukafusp alfa or RO6874281) is a novel, monomeric, immune-cytokine designed to overcome the limitations of wild-type IL2 by selectively promoting immune responses in the tumor microenvironment (4). FAP-IL2v consists of a single IL2v moiety with abolished CD25 binding capacity, fused with a human IgG1 antibody directed against FAP, a type II transmembrane glycoprotein with proteolytic activity (4). FAP, which is rarely expressed in healthy adult tissue, is involved in tissue remodeling processes and has been shown to be highly expressed on the surface of cancer-associated fibroblasts in the stroma of most human solid tumors, including melanoma (5–8). FAP-overexpressing fibroblasts contribute to the generation of a tumor microenvironment that enhances tumor growth and invasiveness (9, 10). In line with findings in other cancers, FAP-positive melanoma-associated fibroblasts have been characterized to play an immunosuppressive role, which supports the potential of an FAP-directed therapy in melanoma (11, 12).

FAP-IL2v was less potent than wild-type FAP-IL2 in activating immunosuppressive regulatory T cells (Treg) *in vitro* (4). Through activation of CD8^+^ T cells and NK cells, FAP-IL2v significantly enhanced the activity of several therapeutic antibodies in preclinical models, including those that mediate checkpoint inhibition via PD-L1 (4). Here, we report results of a clinical phase Ib study investigating the combination of FAP-IL2v with the anti–PD1 monoclonal antibody pembrolizumab for the treatment of patients with mainly anti–PD-1–resistant malignant melanoma.

## Materials and Methods

### Study design

Study BP41054 (NCT03875079) was an open-label, multicenter, phase Ib clinical study to evaluate the safety, tolerability, and antitumor activity of FAP-IL2v in combination with pembrolizumab. Other objectives included the assessment of pharmacodynamic (PD) effects, the pharmacokinetics (PK) of FAP-IL2v, and the formation of anti-drug antibodies (ADA) against FAP-IL2v and pembrolizumab.

The study was conducted in accordance with the Declaration of Helsinki, the International Council for Harmonisation Good Clinical Practice guidelines, and all applicable local and national laws. The Institutional Review Board at each study site approved the study. All patients provided written informed consent.

### Study treatment

Patients first received 200 mg pembrolizumab as a 30-minute intravenous infusion, followed by a 30-minute (if well tolerated, otherwise 2-hour) intravenous infusion of 10 mg FAP-IL2v. The dose of 10 mg FAP-IL2v was selected based on clinical experience, clinical pharmacology results, and biomarker profiles from the evaluation of FAP-IL2v as monotherapy for which the maximum tolerated dose was reached at 15/20 mg with an up-titration regimen (13). Although pembrolizumab was given once every 3 weeks (Q3W) throughout, two schedules were investigated for FAP-IL2v: Either FAP-IL2v was administered on a 3-weekly basis throughout (Q3W schedule), or a 3-week induction phase with weekly (QW) administration preceded a maintenance phase with Q3W dosing (QW/Q3W schedule) to test whether a more intense schedule with weekly FAP-IL2v induction would provide an added benefit over Q3W administration. Both schedules were tested in the safety run-in part of the study before initiation of the extension. The patients were initially randomized to the Q3W or QW/Q3W extension cohorts; however, the QW/Q3W cohort was closed after enrollment of 22 patients, whereas the Q3W cohort continued and enrolled 45 patients overall. The more intense QW/Q3W regimen was abandoned based on considerations related to tolerability, convenience, and potential treatment benefits.

CPI-experienced patients were enrolled in the two extension cohorts for the Q3W and QW/Q3W schedules. CPI-naïve patients were enrolled in the safety run-in; however, an extension planned for CPI-naïve patients was deprioritized and not initiated.

Premedication with antihistamines, antipyretics, an antiemetic, and hydration with 500 mL crystalloid fluid was recommended prior to each infusion. Any infusion-related reactions (IRR) were managed according to protocol-specified guidelines. Similarly, events of liver enzyme elevation were also managed according to guidelines specified in the protocol.

For the management of isolated episodes of fever (no other signs and symptoms), investigators provided guidance to patients who were required to regularly check their body temperature during the days after each administration of FAP-IL2 and take early intervention with standard antipyrexia treatments (e.g., paracetamol and nonsteroidal anti-inflammatory drugs), as per standard practice.

### Study population

Patients ≥18 years were eligible if they had unresectable stage III or stage IV cutaneous or mucosal (only when CPI-naïve) melanoma based on the American Joint Committee on Cancer Staging Manual version 8.0; measurable disease, as defined by RECIST version 1.1; Eastern Cooperative Oncology Group performance score 0 or 1; and life expectancy of ≥12 weeks. For CPI-naïve patients, no prior treatment for advanced disease was allowed; CPI-experienced patients (only cutaneous melanoma) had to have ≥2 cycles of anti–PD-1 antibody therapy, with disease progression within 12 weeks of the last dose. A complete list of eligibility criteria and the representativeness of the study population are included in Supplementary Methods S1 and Supplementary Table S1, respectively.

### Assessments

All adverse events (AE) were graded according to NCI “Common Terminology Criteria for Adverse Events” version 5.04 (CTCAE). PD assessments were carried out in whole blood samples and fresh or archival tumor biopsies, with samples analyzed centrally. Antibodies used for IHC included CD3 (clone 2GV6, Ventana, cat. # 05278422001; RRID: AB_2335978), PRF (clone 5B10, Abcam, cat. # ab89821; RRID: AB_2042606), Ki-67 (clone 30-9, Ventana, cat. # 05278384001; RRID: AB_2631262), CD8 (clone SP239, Ventana, cat. # 09780041001; RRID: not available), FOXP3 (clone 236A-E7, Abcam, cat. # ab20034; RRID: AB_445284), FAP (clone SP325, Spring Biosciences, also known as Abcam, ab227703; RRID: AB_3106978), KRT (clone AE1/AE3/PCK26, Ventana, cat. # 05267145001; RRID: not available), and VENTANA PDL1 (SP264) assay. For flow cytometry, the Q-TBNK Assay Flow Cytometry Panel was used with the following antibodies from BD Biosciences: CD19 (fluorochrome BV421, clone HIB19, cat. # 562440, RRID: AB_11153299), CD4 (fluorochrome BV510, clone SK3, cat. # 659454, RRID: AB_2870492), CD3 (fluorochrome FITC, clone SK7, cat. # 345764, RRID: AB_2916364), CD16 (fluorochrome PE, clone B73.1, cat. # 332779, RRID: AB_2868628), CD56 (fluorochrome PE, clone NCAM16.2, cat. # 345812, RRID: AB_2629216), CD45 (fluorochrome PerCP-Cy5.5, clone 2D1, cat. # 332784, RRID: AB_2868632), CD8 (fluorochrome APC, clone SK1, cat. # 345775, RRID: AB_2868803), CD14 (fluorochrome APC-H7, clone MpH9, cat. # 641394, RRID: AB_1645725), CD57 (fluorochrome APC, clone NK-1, cat. # 560845, RRID: AB_10563760), Ki-67 (fluorochrome AF480, clone B56, cat. # 561165, RRID: AB_10611866), and HLA-DR (fluorochrome PE-Cy7, clone G46-6, cat. # 560651, RRID: AB_1727528).

PK analyses were carried out on serum samples. Validated assays were used to detect ADAs against FAP-IL2v and pembrolizumab. Tumor response was evaluated according to RECIST version 1.1. Details on the assessment are provided in the Supplementary Methods S1.

### Statistical analysis

The safety analysis included all patients who received at least one dose of study treatment. Patients were evaluable for antitumor activity if they received at least one dose of study treatment and had at least one postbaseline tumor assessment or had clinical progression before that. Statistical analyses were performed using SAS version 9.4. PK parameters for FAP-IL2v were determined via noncompartmental analysis.

### Data availability

For up-to-date details on Roche’s Global Policy on the Sharing of Clinical Information and how to request access to related clinical study documents, visit https://go.roche.com/data_sharing. Anonymized records for individual patients across more than one data source external to Roche cannot, and should not, be linked due to a potential increase in risk of patient reidentification.

## Results

### Patient disposition and characteristics

Eighty-three patients were enrolled between June 24, 2019, and July 14, 2022, at 23 sites in seven countries. For the safety run-in part, eight patients were enrolled in each of two cohorts (Supplementary Fig. S1) in which FAP-IL2v was either administered Q3W from the start (Q3W schedule) or after a 3-week induction phase with QW administration (QW/Q3W schedule), together with pembrolizumab Q3W. In the extension part, 67 patients were treated: 45 in the Q3W cohort and 22 in the QW/Q3W cohort. The more intensive QW/Q3W dosing regimen was halted after enrollment of 22 patients based on considerations of tolerability, convenience, and potential benefit.

Seventy-five (90.4%) patients had received previous CPI treatment, alone and/or in combination with other treatments such as CTLA-4 or LAG-3 inhibitors (Supplementary Table S2). Patients in the extension part had on average approximately two prior CPI treatments (Supplementary Table S2). Eight patients were CPI-naïve, and seven were enrolled in the Q3W cohort of the safety run-in (Supplementary Fig. S1). Of the 75 CPI-experienced patients, 46 received Q3W, and 29 received QW/Q3W treatment.

The median treatment duration was 4.4 and 2.9 months in the Q3W and QW/Q3W cohorts of the safety run-in, respectively, and 1.7 and 3.1 months in the Q3W and QW/Q3W extension cohorts, respectively.

Thirteen of 16 patients of the safety run-in and 66 of 67 patients of the extension part discontinued treatment, mostly due to progressive disease (Supplementary Fig. S1).

The median age was 58 years at baseline (Table 1). Most patients had metastatic disease, and 58% had elevated lactate dehydrogenase levels.

**Table 1:** Baseline patient characteristics

	Safety run-in		Extension		Total
	Q3W	QW/Q3W	Q3W	QW/Q3W	
	*n* = 8	*n* = 8	*n* = 45	*n* = 22	*n* = 83
Median age (min–max), years	50.5 (21–72)	52.0 (28–67)	61.0 (35–86)	59.5 (23–84)	58.0 (21–86)
Sex, *n* (%)					
Male	3 (37.5%)	3 (37.5%)	28 (62.2%)	12 (54.5%)	46 (55.4%)
Female	5 (62.5%)	5 (62.5%)	17 (37.8%)	10 (45.5%)	37 (44.6%)
Race, *n* (%)					
White	7 (87.5%)	8 (100%)	37 (82.2%)	18 (81.8%)	70 (84.3%)
Other/unknown	1 (12.5%)	0	8 (17.8%)	4 (18.2%)	13 (15.7%)
ECOG PS at screening, *n* (%)					
0	6 (75.0%)	5 (62.5%)	25 (55.6%)	12 (54.5%)	48 (57.8%)
1	2 (25.0%)	3 (37.5%)	20 (44.4%)	10 (45.5%)	35 (42.2%)
Number of previous lines of systemic therapy, *n* (%)					
0	7 (87.5%)	0	3 (6.7%)	1 (4.5%)	11 (13.3%)
1	1 (12.5%)	5 (62.5%)	24 (53.3%)	13 (59.1%)	43 (51.8%)
2	0	1 (12.5%)	4 (8.9%)	2 (9.1%)	7 (8.4%)
≥3	0	2 (25.0%)	14 (40.0%)	8 (36.4%)	22 (26.5%)
Metastasis stage, *n* (%)					
Missing	4 (50.0%)	3 (37.5%)	2 (4.4%)	0	9 (10.8%)
M0	0	0	1 (2.2%)	1 (4.5%)	2 (2.4%)
M1	1 (12.5%)	1 (12.5%)	7 (15.6%)	7 (31.8%)	16 (19.3%)
M1A	2 (25.0%)	1 (12.5%)	2 (22.2%)	0	13 (15.7%)
M1B	0	1 (12.5%)	5 (11.1%)	1 (4.5%)	7 (8.4%)
M1C	1 (12.5%)	2 (25.0%)	17 (37.8%)	12 (54.5%)	32 (38.6%)
M1D	0	0	3 (6.7%)	1 (4.5%)	4 (4.8%)
Number of target and nontarget lesions per patient, *n* (%)					
1–3	2 (25.0%)	3 (37.5%)	11 (24.4%)	5 (22.7%)	21 (25.3%)
**≥**4	6 (75.0%)	5 (62.5%)	34 (75.6%)	17 (77.3%)	62 (74.7%)
Mutated *BRAF*, *n* (%)	7 (87.5%)	2 (25.0%)	10 (22.2%)	5 (22.7%)	24 (28.9%)
Positive PD-L1 status^a^, *n* (%)	1 (12.5%)	4 (50.0%)	11 (24.4%)	8 (36.4%)	24 (28.9%)
LDH level ≥ upper limit, *n* (%)	3 (37.5%)	3 (37.5%)	27 (60.0%)	15 (68.2%)	48 (57.8%)

Abbreviations: ECOG PS, Eastern Cooperative Oncology Group Performance Status; LDH, lactate dehydrogenase.

a Based on Ventana SP263 assay and combined algorithm to assess tumor and immune cell expression per tumor area (tumor area positive, cut-off 5%; ref. 30).

### Safety

Overall, all but one patient in the Q3W extension part of the study experienced at least one AE related to FAP-IL2v (Table 2).

**Table 2:** Overview of AEs

	Safety run-in		Extension		Total
MedDRA preferred term, *n* (%)	Q3W	QW/Q3W	Q3W	QW/Q3W	
	*n* = 8	*n* = 8	*n* = 45	*n* = 22	*n* = 83
Patients with at least 1					
AE	8 (100)	8 (100)	45 (100)	22 (100)	83 (100)
AE leading to FAP-IL2v dose modification/interruption	0	1 (12.5)	7 (15.6)	7 (31.8)	15 (18.1)
AE leading to treatment discontinuation	0	0	1 (2.2)	0	1 (1.2)
Serious AE	4 (50.0)	3 (37.5)	24 (53.3)	10 (45.5)	41 (49.4)
Serious AE related to FAP-IL2v	2 (25.0)	1 (12.5)	14 (31.1)	8 (36.4)	25 (30.1)
Grade 3 AE	7 (87.5)	4 (50.0)	20 (44.4)	14 (63.6)	45 (54.2)
Grade 4 AE	2 (25.0)	0	8 (17.8)	4 (18.2)	14 (16.9)
AE with fatal outcome	0	0	3 ( 6.7)	1 ( 4.5)	4 ( 4.8)
AE related to FAP-IL2v with fatal outcome	0	0	0	0	0

Most common (in ≥20% total patients) AEs related to FAP-IL2v	Grade		Grade		Grade		Grade		Grade	
	1/2	3/4	1/2	3/4	1/2	3/4	1/2	3/4	1/2	3/4
Patients with at least 1 FAP-IL2v-related AE	1 (12.5)	7 (87.5)	4 (50.0)	3 (37.5)	45 (100)	17 (37.8)	10 (45.5)	12 (54.5)	44 (53.0)	39 (47.0)
Pyrexia	6 (75.0)	0	6 (75.0)	0	19 (42.2)	1 (2.2)	12 (54.5)	0	43 (51.2)	1 (1.2)
IRR	3 (37.5)	0	2 (25.0)	1 (12.5)	19 (42.2)	3 (6.7)	9 (40.9)	1 (4.5)	33 (39.8)	5 (6.0)
Chills	5 (62.5)	0	4 (50.0)	0	16 (35.6)	0	8 (36.4)	0	33 (39.8)	0
Nausea	5 (62.5)	1 (12.5)	3 (37.5)	0	13 (28.9)	1 (2.2)	7 (31.8)	0	28 (33.3)	2 (2.4)
Aspartate aminotransferase increased	5 (62.5)	1 (12.5)	4 (50.0)	0	12 (26.7)	2 (4.4)	4 (18.2)	1 (4.5)	25 (29.8)	4 (4.8)
Asthenia	2 (25.0)	0	4 (50.0)	0	13 (28.9)	0	6 (27.3)	1 (2.2)	25 (29.8)	1 (1.2)
Alanine aminotransferase increased	5 (62.5)	1 (12.5)	3 (37.5)	0	11 (24.4)	2 (4.4)	1 (4.5)	2 (9.1)	20 (23.8)	5 (6.0)
Lymphopenia	0	5 (62.5)	0	2 (25.0)	0	8 (17.8)	0	4 (18.2)	0	19 (22.9)
γ-Glutamyltransferase increased	12 (12.5)	5 (62.5)	1 (12.5)	1 (12.5)	5 (11.1)	1 (2.2)	3 (13.6)	0	10 (11.9)	7 (8.4)

IL2 class-specific AEs following FAP-IL2v treatment included pyrexia (59%), abnormal liver function test (58%), IRR (46%), edema (16%), and capillary leak syndrome (2%).

The most common FAP-IL2v–related AEs included pyrexia (53%), IRR (46%), chills (40%), nausea (36%), elevated aspartate aminotransferase (35%), asthenia (31%), and elevated alanine aminotransferase (ALT, 30%; Table 2) with a median duration for any grade events of 2 days (pyrexia, IRR, and chills), 6.5 days (nausea; grade 3/4: 2 days), 14 days (elevated ALT/aspartate aminotransferase; grade 3/4: 7 days), and 63.5 days (asthenia/fatigue, grade 3/4: 11 days). The majority of these events occurred after the first infusion and mostly within 24 hours of the infusion. The most common FAP-IL2v–related AEs with a grade 3/4 severity were lymphopenia (23%), with a median duration of 4 days, followed by elevated γ-glutamyltransferase (8%), elevated ALT (6%), and IRR (6%; Table 2).

One patient (1.2%) discontinued treatment due to an AE of intracranial hemorrhage. There were 40 serious AEs related to FAP-IL2v in 25 patients (30.1%), and the most common events were IRR (7%) and pyrexia and cytokine release syndrome (4% each).

No deaths considered related to treatment with FAP-IL2 were reported. Overall, 21 deaths were reported: 15 due to progressive disease and 4 due to AEs, including COVID-19, COVID-19/pneumonia, cerebrovascular accident, and disease-related worsening of dyspnea. Two deaths were due to an unknown cause; the study drug was discontinued due to progressive disease in both patients who died 120 and 235 days after the last dose, respectively.

### PK and immunogenicity

The PK analysis of FAP-IL2v in this study demonstrated similar results to that after administration as monotherapy, coadministration with pembrolizumab did not alter the PK profile of FAP-IL2v. FAP-IL2v concentrations exhibited nonlinear elimination typical of target-mediated drug disposition with the expansion of the target pool, consistent with clearance via a nonlinear pathway and via a linear pathway.

A total of 79% and 31% of patients had at least one postdose sample with positive ADA titers in the QW/Q3W and in the Q3W schedule, respectively. The median time to the observation of positive ADA titers was 45 and 63 days in the QW/Q3W and Q3W schedules, respectively. No strong reduction in C_max_ was observed despite the presence of ADA, and no association between positive ADA titers and clinical activity was seen.

### Antitumor activity

**Figure 1:**
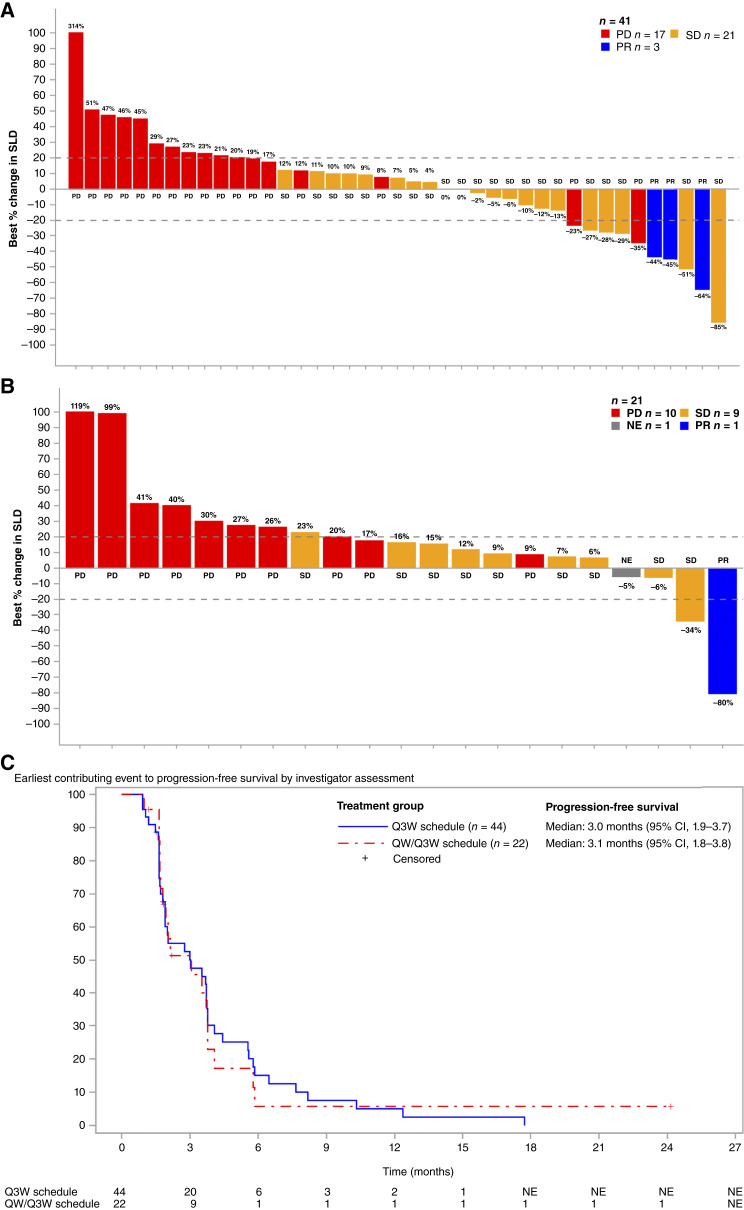
Antitumor activity following treatment with FAP-IL2v of CPI-experienced patients in the extension part with at least one postbaseline tumor assessment (*n* = 62). Best percentage change in the sum of diameters of target lesion from baseline (column color indicates confirmed best overall response) in CPI-experienced patients in the Q3W extension part (*n* = 41; **A**) and in the QW/Q3W extension part (*n* = 21; **B**). **C,** Kaplan–Meier estimate of progression-free survival in patients in the Q3W (blue) and QW/Q3W (red) extension part. Tick marks on the Kaplan–Meier plot show censoring of the data at the last time the patient was known to be alive. NE, not evaluable; PD, progressive disease; SD, stable disease; SLD, sum of longest diameters.

The objective response rate (ORR) was 8.5% [95% confidence interval (CI): 4.20–16.59] for the 82 evaluable patients (Supplementary Table S3). For the seven CPI-naïve patients in the Q3W cohort of the safety run-in, the ORR was 29% (2/7), with one patient achieving a complete response (CR) and another patient having a partial response (PR). Of the 74 CPI-experienced patients enrolled in the other cohorts, 5 (6.5%) achieved PR as best overall response (Supplementary Table S3), four of whom were in the extension cohorts (Fig. 1A and B). The characteristics of the total of seven patients who had a response are summarized in Supplementary Table S4.

A total of 35 patients (42.7%) had stable disease as best overall response. The median progression-free survival (mPFS) was 3.1 months (95% CI, 1.8–3.8) in patients on the Q3W schedule and 3.0 months (95% CI, 1.9–3.7) in patients on the QW/Q3W schedule (Fig. 1C).

### PD/biomarker results

**Figure 2:**
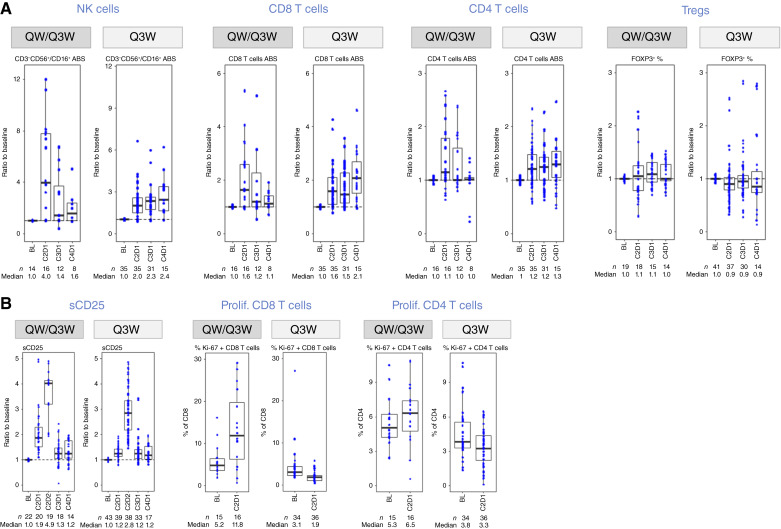
Pharmacodynamic results in peripheral blood. Peripheral blood collected at baseline and various on-treatment time points (e.g., C2D1, C3D1, and C4D1) was assessed for changes in key immune cell subsets such as NK cells, CD8 T cells, CD4 T cells, and Tregs (**A**) and markers for immune cell activation (sCD25) and proliferation (Ki-67^+^; **B**). BL, baseline; C, cycle; D, day.

For all patients enrolled in the extension part, PD changes were assessed in peripheral blood collected in initial treatment cycles. Paired baseline and on-treatment biopsies, most of which could be taken from the same tumor lesion, were available for only a subset of patients (*n* = 27). Consistent with the proposed mode of action, early and marked increases in NK and CD8 T cells in the periphery were observed (Fig. 2A). Compared with the Q3W schedule, the more intense QW/Q3W schedule induced stronger PD effects in patients, particularly for NK cells (two- vs. fourfold induction), whereas the trend was less clear for CD8 T cells. CD4 T cells also slightly increased (up to 1.3-fold induction), whereas the proportion of Tregs overall remained unchanged, as observed in other studies and cohorts (14, 15).

Circulating sCD25, a marker of systemic NK- and T-cell activation, showed an early and transient increase that was also more pronounced for the QW/Q3W (4.9-fold induction) than Q3W schedule (2.8-fold induction; Fig. 2B). T-cell expansion was driven by increased cellular proliferation (Ki-67), which was more pronounced for CD8 T cells but, due to its transient nature, could only be detected in the QW/Q3W schedule (in which the assessment was 1 week after dosing vs. 3 weeks for the Q3W schedule).

**Figure 3:**
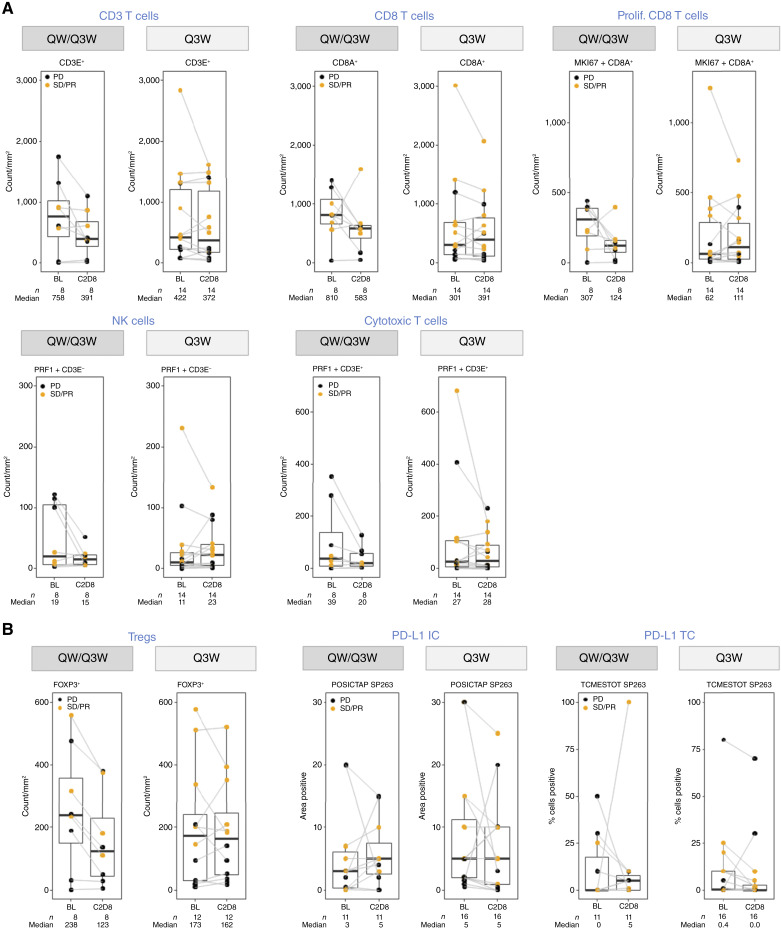
Pharmacodynamic results in tumor tissue. Tumor tissue from biopsies taken at baseline and on treatment (C2D8) was assessed for changes in TIL density with respect to CD3 T cells, CD8 T cells, proliferating CD8 T cells, NK cells, and cytotoxic T lymphocytes (**A**) and Tregs as well as PD-L1 expression on immune and tumor cells (Ventana SP263 assay; **B**). For these exploratory PD assessments, the investigator assessment of tumor response was used. BL, baseline; C, cycle; D, day; IC, immune cell; PD, progressive disease; SD, stable disease; TC, tumor cell.

In tumor tissue, the combination of FAP-IL2v and pembrolizumab did not induce consistent on-treatment changes in key tumor-infiltrating lymphocyte (TIL) populations; a mixed pattern was observed in both schedules (Fig. 3A and B). Across TIL subsets, there was an unexpected trend for decreased TIL levels on treatment for the more intense QW/Q3W schedule. PD-L1 expression was detected at various levels on tumor and immune cells with a trend for higher expression on treatment (Fig. 3B). PD effects were observed in responding and nonresponding patients, and the levels of preexisting tumor infiltration were high and variable, possibly because all patients included in the analysis were previously exposed to CPI treatment.

## Discussion

There are very few effective options for patients with advanced melanoma who do not benefit from first-line treatment with CPIs (2). This phase Ib study explored whether the immune cell activation triggered by FAP-IL2v improves the clinical activity of pembrolizumab in patients with anti–PD-1–resistant melanoma. A schedule that included a 3-week induction phase with QW administration of FAP-IL2v prior to Q3W maintenance dosing was abandoned in favor of Q3W dosing throughout because it was less convenient for patients, had no benefit in terms of clinical activity, and had worse tolerability as indicated by a higher proportion of patients with grade 3 AEs (63.6% vs. 44.4%) or FAP-IL2v dose modification or interruption (31.8% vs. 15.6%).

The safety profile of FAP-IL2v in combination with pembrolizumab is manageable and consistent with the known profiles. The safety profile seemed to be better than that of aldesleukin, an IL2 “wild-type” analog approved for the treatment of metastatic renal cell carcinoma and metastatic melanoma that is associated with the frequent occurrence of capillary leak syndrome necessitating close patient monitoring in a hospital setting (16). The overall safety profile was similar across treatment combinations and cancer types (13–15, 17).

Two of the seven CPI-naïve patients (6.5%) in the Q3W cohort of the safety run-in had a response: one CR and one PR. The ORR of the total population was 8.5%; the mPFS was approximately 3 months, which seems similar to the results of a phase I/IIa study of nivolumab and relatlimab in patients with advanced melanoma with prior progression on anti–PD-1–/PD-L1–containing regimens (ORR: 9.2%–12.0%, mPFS: 2.1–3.2 months; ref. 18). Better outcomes in a similar patient population were seen in phase II studies investigating a tyrosine kinase inhibitor (lenvatinib) and CPI (pembrolizumab; ORR: 21%; ref. 19), a CTLA-4 inhibitor (ipilimumab) and PD-1 CPI nivolumab (ORR: 28%; ref. 20), and an autologous TIL therapy (lifileucel; ORR: 36%; ref. 21).

Consistent with the mode of action of IL2 treatment (22), FAP-IL2v in combination with pembrolizumab preferentially expanded NK and CD8 T cells, whereas the effect was lower on CD4 T cells and absent on Tregs overall. This is in contrast to the preferential expansion of Tregs among CD4 T cells typically observed in patients treated with the “wild-type” IL2 (22), which can potentially lead to impaired antitumor immune responses acknowledging the complex biology of Tregs, which is highly context-specific (23). The observed PD effects were more pronounced for the more intense QW/Q3W schedule, especially for NK cells. Although PD effects seen in peripheral blood were consistent with other studies, treatment combinations, and indications (14, 17), the combination of FAP-IL2v plus pembrolizumab did not lead to increased TIL infiltration and activation, which may reflect the low clinical activity seen in patients. In addition, baseline variability was high, possibly due to prior CPI treatment and multiple acquired resistance mechanisms (24), and almost 60% of the patients had high baseline levels of lactate dehydrogenase, which is a negative predictor of prognosis (25).

In contrast to the combination of FAP-IL and pembrolizumab in this study in anti–PD-1–resistant melanoma, overall positive signals of activity were observed with the combination of FAP-ILv2 plus atezolizumab in CPI-naïve patients with oesophageal cancer (15), cervical squamous cell carcinoma (14), and renal cell carcinoma (17). It is not yet clear why the combination of FAP-IL2v with checkpoint inhibition did not lead to a meaningful antitumor response in this study. The reasons are presumably multifactorial and may include loss of tumor antigen expression or alterations in antigen presentation capacities and upregulation of immuno-suppressive surface molecules or other suppressive mediators leading to immune cell exhaustion, resulting in anti–PD-1 resistance and IL2 unresponsiveness. A bispecific PD-1–IL2v molecule designed to deliver IL2v *in cis* to PD-1^+^ immune cells without CD25 binding resulted in an improved efficacy in chronic infection and cancer models (26, 27).

The pegylated IL2 cytokine prodrug bempegaldesleukin also did not improve clinical outcomes when given in combination with the PD-1 CPI nivolumab in previously untreated patients with advanced melanoma (28). The reasons for the failure are not clear; increases in absolute lymphocyte count and sCD25 demonstrated that bempegaldesleukin was biologically active. Thus, the full potential of targeting the IL2 pathway for the treatment of advanced melanoma remains to be exploited.

In conclusion, although the safety profile of FAP-IL2v in combination with pembrolizumab was manageable, the combination showed no added benefit for the treatment of CPI-experienced patients with melanoma who had progressive disease while receiving anti–PD-1 CPI therapy in the previous line of treatment. Signs of biological activity observed in peripheral blood did not translate into meaningful changes in the tumor microenvironment, and the low clinical activity indicates that resistance to PD-1 CPI cannot be overcome by the addition of FAP-IL2v. The low ORR may only represent the antitumor activity of FAP-IL2v in this population. Considering the PD effects, the addition of FAP-IL2v to pembrolizumab or nivolumab may be of greater value in previously untreated patients although the benefit-risk profile of FAP-IL2v may make it challenging to investigate this in the first-line setting.

The sponsor decided to discontinue the development of FAP-IL2v for strategic reasons; however, alternative approaches for simultaneous targeting of PD-1 and IL2-receptors (e.g., on PD-1^+^ T stem–like cells) and the capability of IL2 to rewire exhausted CD8 T cells (29) warrant further investigation in immunogenic, T cell-infiltrated active tumor types such as melanoma and others.

## Supplementary Material

Figure S1 Study flow chart

Methods S1 Supplementary methods

Table S1 Representativeness of study participants

Table S2 Summary of prior therapy

Table S3 Antitumor activity per RECIST version 1.1

Table S4 Characterization of patients with complete or partial response
