# Supplementary material for: Phase Ib Study of Immunocytokine Simlukafusp Alfa (FAP-IL2v) Combined with Pembrolizumab for Treatment of Advanced and/or Metastatic Melanoma
Source: Cancer Res Commun. 2025 Feb 24;5(2):358–68. doi: 10.1158/2767-9764.CRC-24-0601 (PMC11848832; doi:10.1158/2767-9764.CRC-24-0601)
Supplement: Figure S1 — Study flow chart [file crc-24-0601_figure_s1_suppsf1.docx]

**Supplementary Figure S1** Study flow chart


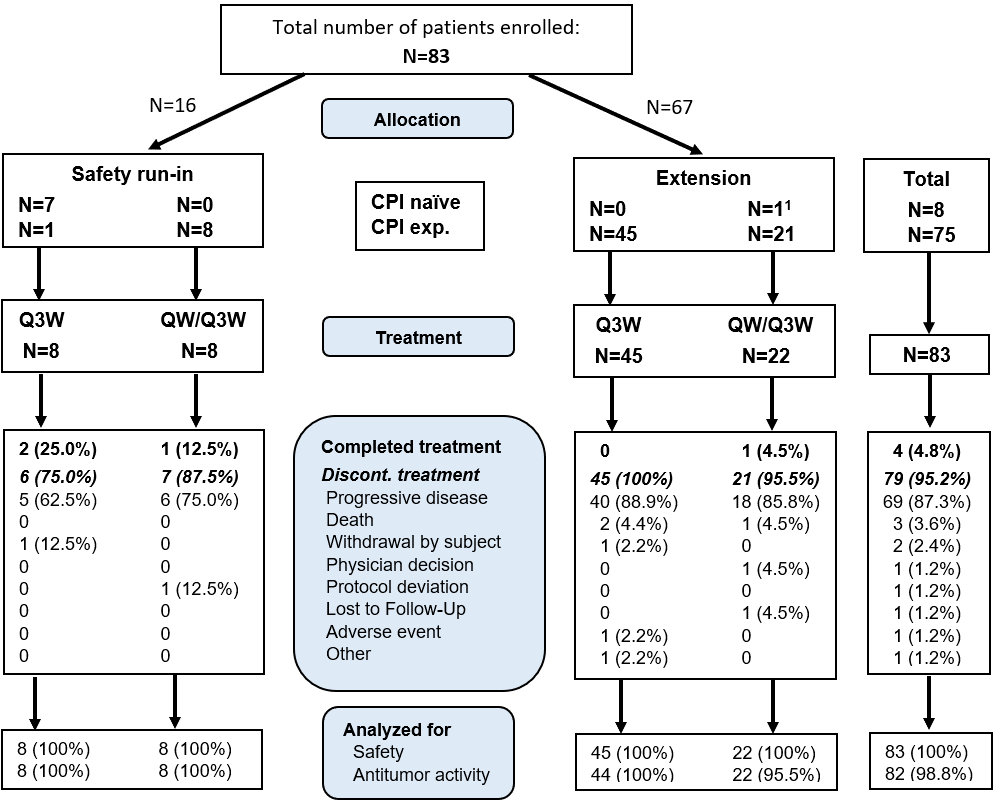


^1^ One CPI-naïve patient was erroneously enrolled in the extension part of the study.

Abbreviations: CPI, checkpoint inhibitor; once every week; QW, once every 3 weeks.
