# Supplementary material for: Phase Ib Study of Immunocytokine Simlukafusp Alfa (FAP-IL2v) Combined with Pembrolizumab for Treatment of Advanced and/or Metastatic Melanoma
Source: Cancer Res Commun. 2025 Feb 24;5(2):358–68. doi: 10.1158/2767-9764.CRC-24-0601 (PMC11848832; doi:10.1158/2767-9764.CRC-24-0601)
Supplement: Methods S1 — Supplementary methods [file crc-24-0601_methods_s1_suppsm1.docx]

**Supplementary methods**

**Eligibility criteria for the clinical study**

***Inclusion criteria***

Patients had to meet the following criteria for study entry:

1. Signed informed consent.
2. Age ≥ 18 years.
3. Histologically confirmed unresectable stage III or stage IV cutaneous or mucosal melanoma (The American Joint Committee on Cancer [AJCC] v8.0).
4. Known BRAF status.
5. CPI-naïve melanoma population:

- Participants with unresectable stage III or stage IV cutaneous or mucosal melanoma who had not received prior treatment for advanced disease. BRAF mutation-positive patients were eligible without prior treatment or after failure of BRAF-directed inhibitor therapy.
- Confirmed at least one non-target tumor lesion to undergo baseline tumor biopsy. If on-treatment biopsies became mandatory, participants had to have sufficient non-target tumor tissue.
- Participants may have had adjuvant treatment with anti-PD-1/PD-L1 or anti-CTLA-4 antibody therapy if it was discontinued at least 6 months prior to Cycle 1 Day 1.

CPI-experienced melanoma population: Participants with unresectable stage III or stage IV cutaneous melanoma. Participants had to have progressed during or after treatment with anti-PD-1 antibody therapy, either as monotherapy or in combination with other agent(s).

- Confirmed at least one non-target tumor lesion to undergo baseline and on-treatment tumor biopsies.
- Disease progression within 12 weeks of the last dose of anti-PD-1 antibody therapy.
- Participants had to have received a minimum of two cycles of anti-PD-1 antibody therapy.
- Eligibility of participants receiving previous treatment with non-approved anti-PD-1 antibodies or combination treatment with non-approved other agent(s) had to be agreed by the Sponsor prior to enrollment.
- The interval between last anti-PD-1 antibody and start of study treatment had to be at least 14 days.
- No intervening anti-cancer therapy, including targeted therapies for BRAF mutation-positive participants, between the last course of anti-PD-1 antibody and the first dose of study treatment was allowed except for local measures (e.g., surgical excision or biopsy, focal radiation therapy).
- Participants that received adjuvant treatment with anti-PD-1/PD-L1 or anti-CTLA-4 antibody therapy could be considered if the relapse occurred during or within 6 months of completion of adjuvant treatment.

1. Measurable disease, as defined by RECIST v1.1.

- Participants had to have at least one measurable lesion not intended to be biopsied.
- Previously irradiated lesions were not to be counted as target lesions unless there had been demonstrated progression in the lesion and no other target lesions were available.
- Lesions that were intended to be biopsied were not to be counted as target lesions.

1. ECOG Performance Status 0 or 1 or Karnofsky performance status (KPS) ≥ 70.
2. Life expectancy of ≥ 12 weeks.
3. Consent to provide an archival tumor tissue sample (if available).
4. Participants were to have adequate cardiovascular function.

- New York Heart Association (NYHA) Heart Failure Stage ≤ 2
- Left ventricular ejection fraction ≥ 50%, as determined by multiple gated acquisition scan (MUGA) or transthoracic echocardiogram (TTE). This applied to participants with cardiovascular conditions in their medical history and all participants >50 years of age
- Baseline corrected QT (QTcF) interval ≤ 470 milliseconds
- Resting systolic blood pressure ≤ 150 mmHg and diastolic blood pressure ≤ 100 mmHg (average of ≥ 3 readings on ≥ 2 sessions)
- Resting heart rate between 45 to 100 bpm

1. Adequate hematological function: neutrophil count of ≥ 1.5 x 10^9^ cells/L, platelet count of ≥ 100,000/L, hemoglobin ≥ 9 g/dL (5.6 mmol/L), lymphocytes ≥ 0.5 x 10^9^ cells/L. Borderline lymphocyte cell counts could be confirmed by a manual count.
2. Adequate liver function, including total bilirubin ≥ 1.5 x upper limit of normal (ULN; direct bilirubin ≥ ULN for participants with total bilirubin levels >1.5 ULN); aspartate aminotransferase (AST), and alanine aminotransferase (ALT) ≥ 2.5 x ULN.

In case of liver metastases, AST and ALT: ≤ 5 x ULN. Eligibility of patients with liver metastases was to be discussed and agreed with the Sponsor if AST and ALT were between 2.5 x and 5 x ULN.

1. Adequate renal function: serum creatinine ≤ 1.5 x ULN or creatinine clearance by Cockcroft Gault formula (see Appendix 6) ≥ 50 mL/min for participants in whom, in the Investigator's judgment, serum creatinine levels did not adequately reflect renal function
2. Participants with unilateral pleural effusion were eligible if they fulfilled both of the following:

- NYHA Class 1
- Forced expiratory volume 1 (FEV1) >70% and forced vital capacity (FVC) >70% of predicted value; participants with lung metastases were to present with DLCO >60% of predicted value

1. Sex

- Male and female participants

The contraception and abstinence requirements were intended to prevent exposure of an embryo to the study treatment. The reliability of sexual abstinence for male and/or female enrollment eligibility needed to be evaluated in relation to the duration of the clinical study and the preferred and usual lifestyle of the participant. Periodic abstinence (e.g., calendar, ovulation, symptothermal, or post ovulation methods) and withdrawal were not acceptable methods of contraception.

- Female participants

A female participant was eligible to participate if she was not pregnant, not breastfeeding, and at least one of the following conditions applied:

- Not a woman of childbearing potential (WOCBP).

OR

- A WOCBP, who: Agreed to remain abstinent (refrain from heterosexual intercourse) or use contraceptive methods that resulted in a failure rate of < 1% per year during the treatment period and for at least 4 months after the last dose of study drug for FAP-IL2v and for at least 4 months after the last dose of pembrolizumab.

Examples of contraceptive methods with a failure rate of < 1% per year included bilateral tubal occlusion, male sterilization, established proper use of hormonal contraceptives that inhibit ovulation, and hormone releasing intrauterine devices.

- Had a negative pregnancy test (serum) within the 7 days before the first study treatment administration.
- Male participants

During the treatment period and for at least 2 months after the last dose of FAP-IL2v, agreement to the following:

- Remained abstinent (refrain from heterosexual intercourse) or used contraceptive measures such as a condom plus an additional contraceptive method that together resulted in a failure rate of < 1% per year, with partners who are WOCBP.
- With pregnant female partners, remained abstinent (refrain from heterosexual intercourse) or used contraceptive measures such as a condom to avoid exposing the embryo.
- Refrained from donating sperm.

***Exclusion criteria***

Participants were excluded from the study if any of the following criteria applied:

Medical Conditions:

1. Rapid disease progression or suspected hyperprogression (as determined by the Investigator) or threat to vital organs or critical anatomical sites (e.g., respiratory failure due to tumor compression, spinal cord compression) requiring urgent alternative medical intervention.
2. Known active central nervous system (CNS) metastases and/or carcinomatous meningitis/leptomeningeal disease:

Participants with previously treated brain metastases could participate provided they

- Were stable (without evidence of progression by computed tomography (CT) or MRI for at least four weeks prior to the first dose of study drug).
- Had no evidence of new or enlarging brain metastases.
- Were off systemic steroids for at least two weeks prior to first study drug administration (anticonvulsants at a stable dosage are allowed).

1. History of treated asymptomatic CNS metastases with any of the following criteria:

- Metastases to brain stem, midbrain, pons, medulla, cerebellum, or within 10 mm of the optic apparatus (optic nerves and chiasm).
- History of intracranial hemorrhage or spinal cord hemorrhage.
- Stereotactic radiation or whole-brain radiation within 28 days before study treatment administration.
- CNS metastases treated by neurosurgical resection or brain biopsy performed within 28 days before study treatment administration.

1. An active second malignancy (exceptions are non-melanoma skin cancer, cervical carcinoma in situ, or prostate carcinoma that is in remission under androgen deprivation therapy for ≥ 2 years, or participants who had a history of malignancy and had been treated with curative intent and the participant was expected to be cured as per Investigator’s assessment).

Other exceptions may have applied and required discussion between the Investigator and the Sponsor.

1. Evidence of significant, uncontrolled concomitant diseases that could have affected compliance with the protocol or interpretation of results, and known autoimmune diseases or other disease with ongoing fibrosis (such as scleroderma, pulmonary fibrosis. and emphysema).
2. Episode of significant cardiovascular/cerebrovascular acute disease within 6 months before study treatment administration, including any of the following: hypertensive crisis/encephalopathy, unstable angina, transient ischemic attack, congestive heart failure without adequate cardiovascular function (for NYHA classification, refer to inclusion criteria), serious cardiac arrhythmia requiring treatment (exceptions are atrial fibrillation, paroxysmal supraventricular tachycardia), history of thromboembolic events (such as myocardial infarction, stroke or pulmonary embolism).
3. Active or uncontrolled infections, including latent tuberculosis.
4. Known HIV infection.
5. Active hepatitis B virus (HBV) or hepatitis C virus (HCV) infection.
6. Severe infection within 4 weeks before study treatment administration, including, but not limited to, hospitalization for complications of infection, bacteremia, or severe pneumonia.
7. History of chronic liver disease or evidence of hepatic cirrhosis.
8. Dementia or altered mental status that prohibited informed consent.
9. History of autoimmune disease, including, but not limited to, systemic lupus erythematosus, rheumatoid arthritis, inflammatory bowel disease, vascular thrombosis associated with antiphospholipid syndrome, Wegener granulomatosis, Sjögren syndrome, Guillain Barré syndrome, multiple sclerosis, vasculitis, or glomerulonephritis with the following exceptions:

- Patients with a history of autoimmune hypothyroidism on a stable dose of thyroid replacement hormone may have been eligible.
- Patients with controlled type 1 diabetes mellitus on a stable insulin regimen may have been eligible.
- Patients with eczema, psoriasis, lichen simplex chronicus, or vitiligo with dermatologic manifestations only (e.g., no psoriatic arthritis) may have been eligible provided that they met the following conditions:
- Rash had to cover less than 10% of the body surface area
- Disease was well controlled at baseline and only required low potency topical steroids
- There were no acute exacerbations of underlying condition within the last 12 months (e.g., not requiring psoralen plus ultraviolet A radiation, methotrexate, retinoids, biologic agents, oral calcineurin inhibitors, high potency, or oral steroids)

1. Adverse events related to any previous radiotherapy, chemotherapy, targeted therapy, CPI therapy or surgical procedure that had not resolved to Grade ≤ 1, except alopecia (any grade) and Grade 2 peripheral neuropathy.
2. History of idiopathic pulmonary fibrosis, pneumonitis (including drug-induced), organizing pneumonia (i.e., bronchiolitis obliterans, cryptogenic organizing pneumonia, etc.), or evidence of active pneumonitis on screening chest CT scan.

History of radiation pneumonitis in the radiation field (fibrosis) is permitted.

1. Bilateral pleural effusion.
2. Severe dyspnea at rest or requiring supplementary oxygen therapy.
3. Concurrent therapy with any other investigational drug (defined as a treatment for which there was currently no regulatory authority-approved indication).
4. Immunomodulating agents:

- Last dose with any of the following agents, for example, etanercept, infliximab, tacrolimus, cyclosporine, mycophenolic acid, alefacept, or efalizumab (or similar agents) < 28 days before study treatment administration.
- Regular immunosuppressive therapy (i.e., for organ transplantation, chronic rheumatologic disease).

1. Treatment with systemic immunosuppressive medications including, but not limited to prednisone, cyclophosphamide, azathioprine, methotrexate, thalidomide, and anti-TNF agents within 2 weeks prior to Cycle 1 Day 1.

Participants who had received acute and/or low-dose systemic immunosuppressive medications (e.g., a one-time dose of dexamethasone for nausea or chronic use of ≤ 10 mg/day of prednisone or dose-equivalent corticosteroid) could be enrolled in the study after discussion with and approval by the Medical Monitor.

The use of inhaled corticosteroids (e.g., fluticasone for chronic obstructive pulmonary disease) was allowed.

The use of oral mineralocorticoids (e.g., fludrocortisone for patients with orthostatic hypotension) was allowed.

Physiologic doses of corticosteroids for adrenal insufficiency were allowed.

1. Radiotherapy within the last 4 weeks before start of study treatment administration, with the exception of limited field palliative radiotherapy.
2. Administration of a live, attenuated vaccine within 4 weeks before Cycle 1 Day 1.
3. Major surgery or significant traumatic injury < 28 days before study treatment administration (excluding fine needle biopsies) or anticipation of the need for major surgery during study treatment.
4. Known hypersensitivity to any of the components of the RO6874281 drug product or pembrolizumab drug product, including but not limited to hypersensitivity to Chinese Hamster Ovary cell products or other recombinant human or humanized antibodies.
5. No prior cytotoxic therapy for unresectable stage III or stage IV disease is permitted.
6. Toxicity from prior anti-PD-1 antibody therapy (including adjuvant treatment) as defined:

- Any history of an immune related Grade 4 adverse event attributed to prior CIT (other than endocrinopathy managed with replacement therapy or asymptomatic elevation of serum amylase or lipase)
- Any history of an immune related Grade 3 adverse event attributed to prior CIT that required permanent discontinuation of the prior immunotherapeutic agent per local prescribing information, European Society for Medical Oncology (ESMO) guidelines, or American Society of Clinical Oncology (ASCO) guidelines
- Adverse events from prior anti-cancer therapy that had not resolved to Grade ≤ 1 except for alopecia, vitiligo, or endocrinopathy managed with replacement therapy
- Patients with asymptomatic elevations of lipase/amylase may have been eligible following discussion with the Medical Monitor
- Immune related adverse events related to prior CIT (other than endocrinopathy managed with replacement therapy or stable vitiligo) that had not resolved to baseline
- Patients treated with corticosteroids for immune related adverse events had to demonstrate absence of related symptoms or signs for ≥ 4 weeks following discontinuation of corticosteroids.

Participant eligibility for treatment with pembrolizumab was to be verified against pembrolizumab labeling documents.

**Study assessments**

***Safety***

Safety assessments included physical examinations, vital signs, clinical laboratory parameters, and electrocardiograms. All adverse events (AEs), regardless of relationship to study treatment, were reported from initiation of study treatment until 3 months after the last dose of study treatment or until the initiation of a post study anti-cancer treatment, whichever occurred first. The severity of AEs was graded according to NCI CTCAE v5.04.

***Pharmacokinetics***

Pharmacokinetic analyses were carried out on serum samples using an acid dissociated homogeneous ELISA method using biotinylated huFAP as capture reagent and digoxigenylated hIL2R along with horseradish peroxidase-conjugated mouse anti-digoxigenin Fab fragments as detection reagents. Validated screening, confirmatory, and titer assays were used to detect ADAs against FAP-IL2v and pembrolizumab before, during, and after treatment.

***Clinical activity***

Response to treatment was assessed using computed tomography scans (or magnetic resonance imaging) of the chest, abdomen, and pelvis at screening and every 8 weeks after study treatment for the first year, and every 12 weeks thereafter until disease progression or treatment discontinuation. Tumor response was evaluated according to RECIST v1.1.

***Pharmacodynamics***

Pharmacodynamic assessments were carried out in whole blood samples and fresh or archival tumor biopsies, with samples analyzed centrally. Paired baseline and on-treatment tumor biopsies were to be taken from the same location whenever possible. Whole blood and plasma samples were collected to analyze changes in key biomarkers related to immune activation. Tumor tissue was subjected to immunohistochemistry for analysis of markers of tumor inflammation and infiltration (e.g., PD-L1, CD8 tumor-infiltrating lymphocytes). Pharmacodynamic changes in peripheral immune cells, cytokines, and inflammatory mediators were assessed at multiple timepoints with focus on the initial cycles of treatment.

Human specimens

All biologic material used in this study and their subsequent evaluations were in accordance with the informed consent agreements obtained from all patients.

Specimen collection

In order to assess key immune cell populations and cytokines, blood and plasma samples were collected from all patients according to the schedule of activities at predefined time points during the course of treatment.

Patients also provided a baseline biopsy sample, either a newly obtained, formalin-fixed tissue specimen or formalin-fixed, paraffin-embedded (FFPE) archival specimen and an on-treatment biopsy sample at the climax of peripheral immune activation (C2D8), for retrospective immunohistochemical (IHC) and genomic biomarker analyses (GEP). Biopsy specimens were sent to a central pathology laboratory (TARGOS, Kassel, Germany), and newly obtained, formalin-fixed tissue specimens were embedded on receipt, sectioned, and stained.

Immune-phenotyping/flow cytometry (whole blood samples)

Key immune cell subsets (T, B, NK cells, activated T, B, NK cells, proliferating T cells, naïve and memory T cells, and regulatory T cells) were evaluated centrally using validated flow cytometry analyses (fluorescence activated cell sorting [FACS] Basic and FACS advanced).

Immunoassay (plasma)

Plasma samples were collected for cytokine (e.g., soluble IL-2R) analysis using a validated enzyme-linked lectin assay (ELLA) and were evaluated centrally. C-reactive protein levels were assessed in local laboratories.

Immunohistochemistry (tumor biopsies)

All biopsy samples were evaluated for quality and sufficient quantity of relevant tissue on haematoxylin and eosin-stained sections by board-certified pathologists. Consecutive sections of FFPE tumor tissues were stained with the following in-house developed IHC assays using Ventana Benchmark XT or Discovery Ultra-automated platforms (Ventana Medical Systems; Tucson, AZ): CD3 (2GV6)/PRF (5B10), Ki67 (30-9)/CD8 (SP239), FOXP3 (236A/E7) and FAP (SP325)/KRT (AE1/AE3&PCK26). All slides were digitized, and the tumor area was manually annotated. The results of automated digital slide analysis were reported as follows: CD3+ T cell, CD3+PRF+ cytotoxic T cell, CD3-PRF+ NK cell, CD8+ T cell, Ki67+/CD8+ proliferating T cell, and FOXP3+ Treg densities as counts/mm^2^ tumor area as well as FAP+ stromal area/mm^2^ tumor area. For PD-L1 assessments, the OptiView DAB VENTANA PDL1 (SP263) procedure on the BenchMark ULTRA instrument was used. Samples were scored for PD-L1 expression on tumor cells and area covered by tumor-infiltrating immune cells, which included macrophages, dendritic cells, and lymphocytes.
