# Supplementary material for: Phase Ib Study of Immunocytokine Simlukafusp Alfa (FAP-IL2v) Combined with Pembrolizumab for Treatment of Advanced and/or Metastatic Melanoma
Source: Cancer Res Commun. 2025 Feb 24;5(2):358–68. doi: 10.1158/2767-9764.CRC-24-0601 (PMC11848832; doi:10.1158/2767-9764.CRC-24-0601)
Supplement: Table S1 — Representativeness of study participants [file crc-24-0601_table_s1_suppst1.docx]

**Supplementary Table S1** Representativeness of Study Participants

| **Cancer type(s)/subtype(s)/stage(s)/ condition** | **Advanced and/or metastatic melanoma** |
| --- | --- |
| **Consideration related to** |  |
| Sex | Melanoma is more common in men. In 2020, the age-standardized global rate of new cases of melanoma of the skin per 100,000 persons was 3.8 in men and 3.0 in females (1). |
| Age | Approximately half of all melanoma cases occur in people over the age of 50, and the median age at diagnosis is 59 years (2). |
| Race/ethnicity | Melanoma is more than 20 times more common in White people than in Black people (3). |
| Geography | In 2020, the highest incidence rates were observed in Australia/New Zealand, followed by Western Europe, North America, and Northern Europe while melanoma was rare in most African and Asian countries (4). |
| Overall representativeness of this study | This phase 1b study was conducted in 83 patients enrolled in Europe and the United States. The median age of the study participants (58 years) was similar to the median age at diagnosis of melanoma reported in the literature. The study enrolled more men than women and predominantly White people, which is consistent with the higher incidence of melanoma in men and people of lighter skin color. |

1. World Cancer Research Fund International. Skin cancer statistics (https://www.wcrf.org/cancer-trends/skin-cancer-statistics/), accessed January 2024. 2020.

2. Age and Risk (https://www.aimatmelanoma.org/melanoma-101/understanding- melanoma/melanoma-risk-factors/age-and-risk/), accessed January 2024.

3. Melanoma Research Alliance. Melanoma Statistics (https://www.curemelanoma.org/about- melanoma/melanoma-101/melanoma-statistics-2), accessed January 2024.

4. Arnold M, Singh D, Laversanne M, Vignat J, Vaccarella S, Meheus F, et al. Global Burden of Cutaneous Melanoma in 2020 and Projections to 2040. JAMA Dermatol. 2022;158:495.
