# Supplementary material for: Phase Ib Study of Immunocytokine Simlukafusp Alfa (FAP-IL2v) Combined with Pembrolizumab for Treatment of Advanced and/or Metastatic Melanoma
Source: Cancer Res Commun. 2025 Feb 24;5(2):358–68. doi: 10.1158/2767-9764.CRC-24-0601 (PMC11848832; doi:10.1158/2767-9764.CRC-24-0601)
Supplement: Table S2 — Summary of prior therapy [file crc-24-0601_table_s2_suppst2.docx]

**Supplementary Table S2** Summary of prior therapy

| **Study part** | **Safety run-in** | | **Extension** | | | | **Total  N=75** |
| --- | --- | --- | --- | --- | --- | --- | --- |
| **Schedule** | **Q3W  N=1** | **QW/Q3W  N=8** | **Q3W  N=45** | | **QW/Q3W  N=21** | |  |
| ***Number of patients with data*** | 1 | 8 | 42 | 20 | | 71 | |
| ***Number of prior CPI therapies, mean (min; max)*** | 1 (1; 1) | 1.2 (1; 2) | 1.9 (1; 8) | 2.1 (1; 9) | | 1.9 (1; 9) | |
| ***Length of last CPI (months), mean (min; max)*** | 5.1  (5.1; 5.1) | 5.8  (0.7; 13.6) | 5.7  (0; 28.2) | 8.0  (0.9; 35.6) | | 6.4  (0; 35.6) | |
| ***Time (months) from last CPI to first study treatment, mean (min; max)*** | 0.9  (0.9; 0.9) | 5.3  (0.7; 35.1) | 2.8  (0.5; 21.5) | 1.8  (0.6; 8.0) | | 2.8  (0.5; 35.1) | |
| ***Best response to last prior CPI therapy**** |  |  |  |  | |  | |
| Patients with CR or PR, *n* (%) | 0 | 3 (37.5) | 4 (8.9) | 1 (4.8) | | 8 (10.6) | |
| Number of patients with CR | 0 | 1 | 1 | 0 | | 2 | |
| PR | 0 | 2 | 3 | 1 | | 6 | |
| SD | 1 | 1 | 9 | 8 | | 19 | |
| PD | 0 | 4 | 24 | 10 | | 38 | |
| NA | 0 | 0 | 5 | 1 | | 6 | |
| ***Patients receiving CPI in*** ***adjuvant setting, n (%)*** |  |  |  |  | |  | |
| Alone or in combination therapy | 0 | 2 (25.0) | 8 (17.8) | 4 (19.0) | | 14 (18.7) | |
| alone | 0 | 1 (12.5) | 5 (11.1) | 4 (19.0) | | 10 (13.3) | |
| with CTLA-4-i | 0 | 1 (12.5) | 3 (6.7) | 0 | | 4 (5.3) | |
| ***Patients receiving CPI in metastatic setting, n (%)*** |  |  |  |  | |  | |
| Alone or in combination therapy | 0 | 8 (100) | 42 (93.3) | 20 (95.3) | | 70 (93.3) | |
| alone | 0 | 3 (37.5) | 31 (68.9) | 17 (81.0) | | 51 (68.0) | |
| with CTLA-4-i | 0 | 4 (50.0) | 20 (44.4) | 7 (33.3) | | 31 (41.3) | |
| with LAG-3-i | 0 | 0 | 3 (6.7) | 6 (28.6) | | 9 (12.0) | |
| with MEK-i | 0 | 1 (12.5) | 1 (2.2) | 0 | | 2 (2.7) | |
| with another drug | 0 | 2 (25.0) | 13 (28.9) | 5 (23.8) | | 20 (26.7) | |
| ***Patients receiving BRAF-i in combination with MEK-i, n %*** |  |  |  |  | |  | |
| adjuvant setting | 0 | 0 | 1 (2.2) | 1 (4.8) | | 2 (2.7) | |
| metastatic setting | 0 | 1 (12.5) | 8 (17.8) | 3 (14.3) | | 12 (16.0) | |

*To be eligible for this study, CPI-experienced patients had to have progressed during or after treatment with anti-PD-1 antibody therapy.

Abbreviations: CPI, checkpoint inhibitor; CR, complete response; i, inhibitor; NA, not available; PR, partial response; SD, stable disease; QW, once every week; QW, once every 3 weeks.
