# Supplementary material for: Phase Ib Study of Immunocytokine Simlukafusp Alfa (FAP-IL2v) Combined with Pembrolizumab for Treatment of Advanced and/or Metastatic Melanoma
Source: Cancer Res Commun. 2025 Feb 24;5(2):358–68. doi: 10.1158/2767-9764.CRC-24-0601 (PMC11848832; doi:10.1158/2767-9764.CRC-24-0601)
Supplement: Table S3 — Antitumor activity per RECIST version 1.1 [file crc-24-0601_table_s3_suppst3.docx]

**Supplementary Table S3** Antitumor activity per RECIST version 1.1

| **Study part** | **Safety run-in** | | | **Extension** | | | **Total  N=82** |
| --- | --- | --- | --- | --- | --- | --- | --- |
| **Schedule CPI status** | **Q3W  exp./naïve^1^ N=8** | | **QW/Q3W exp.  N=8** | **Q3W exp.  N=44** | **QW/Q3W exp.^2^  N=22** | |  |
| **Objective response rate, *n* (%) 95% CI** | 2 (25.0%) 7.15, 59.07 | 1 (12.5%) 2.24, 47.09 | | 3 ( 6.8%) 2.35, 18.23 | 1 ( 4.5%) 0.81, 21.80 | 7 ( 8.5%) 4.20, 16.59 | |
| **Best overall response, *n* (%)** |  |  | |  |  |  | |
| Complete response | 1 (12.5%) | 0 | | 0 | 0 | 1 ( 1.2%) | |
| Partial response | 1 (12.5%) | 1 (12.5%) | | 3 ( 6.8%) | 1 ( 4.5%) | 6 ( 7.3%) | |
| Stable disease | 2 (25.0%) | 3 (37.5%) | | 21 (47.7%) | 9 (40.9%) | 35 (42.7%) | |
| Progressive disease | 4 (50.0%) | 3 (37.5%) | | 19 (43.2%) | 10 (45.5%) | 36 (43.9%) | |
| Missing/not evaluable | 0 | 1 (12.5%) | | 1 ( 2.3%) | 2 ( 9.1%) | 4 ( 4.9%) | |
| **Disease control rate, *n* (%) 95% CI** | 4 (50.0%) 21.52, 78.48 | 4 (50.0%) 21.52, 78.48 | | 24 (54.5%) 40.07, 68.29 | 10 (45.5%) 26.92, 65.34 | 42 (51.2%) 40.59, 61.74 | |

Abbreviations: CI, confidence interval; exp., experienced; QW, once every week; QW, once every 3 weeks.

^1^ Seven CPI-naïve patients and 1 CPI-experienced patient were enrolled into this cohort.

^2^ One CPI-naïve patient was erroneously enrolled in the extension part of the study.
