# Supplementary material for: Phase Ib Study of Immunocytokine Simlukafusp Alfa (FAP-IL2v) Combined with Pembrolizumab for Treatment of Advanced and/or Metastatic Melanoma
Source: Cancer Res Commun. 2025 Feb 24;5(2):358–68. doi: 10.1158/2767-9764.CRC-24-0601 (PMC11848832; doi:10.1158/2767-9764.CRC-24-0601)
Supplement: Table S4 — Characterization of patients with complete or partial response [file crc-24-0601_table_s4_suppst4.docx]

**Supplementary Table S4** Characterization of patients with complete or partial response

| **Sex, age** | **CPI exp.** | **prior lines metastatic treatment** | **Elevated LDH at BL (U/L) ^1^** | **PD-L1 positivity^2^** | **Liver metastasis** | **FAP-IL2v cohort** | **Response  (start)** | **Response duration** |
| --- | --- | --- | --- | --- | --- | --- | --- | --- |
| F, 72y | **-** | - | **-** (145) | + | - | Part 1 Q3W | CR (Day 57) | >645 days |
| F, 84y | **+** | 1 | **+** (259) | + | - | Part 3 QW/Q3W | PR (Day 119) | >617 days |
| M, 44y | **+** | 1 | **+** (7499) | + | + | Part 1 QW/Q3W | PR (Day 115) | >590 days |
| F, 49y | **-** | - | **+** (500) | - | + | Part 1 Q3W | PR (Day 117) | >420 days |
| M, 77y | **+** | 1 | **-** (175) | NA | - | Part 3 Q3W | PR (Day 50) | 265 days |
| F, 41y | **+** | - | **+** (228) | + | - | Part 3 Q3W | PR (Day 64) | 115 days |
| F, 35y | **+** | 4 | **-** (159) | + | - | Part 3 Q3W | PR (Day 117) | 117 days |

BL, baseline; exp., experienced; CR, complete response; F, female; LDH, lactate dehydrogenase; M = male; NA, not available; PR, partial response; QW, once every week; Q3W, once every 3 weeks; y, years.

^1^ Per the protocol, the upper limit of the normal range was defined as 190 U/L for LDH

^2^ Measured by VENTANA PD-L1 (SP263) assay
